# Deficits in the miRNA-34a-regulated endogenous TREM2 phagocytosis sensor-receptor in Alzheimer's disease (AD); an update

**DOI:** 10.3389/fnagi.2014.00116

**Published:** 2014-06-17

**Authors:** Surjyadipta Bhattacharjee, Yuhai Zhao, Walter J. Lukiw

**Affiliations:** Departments of Neurology, Neuroscience and Ophthalmology, Louisiana State University Neuroscience Center and Health Sciences CenterNew Orleans, LA, USA

**Keywords:** Alzheimer's disease, miRNA-34a, TREM2, phagocytosis, innate-immune response, amyloidosis

One characteristic feature of Alzheimer's disease (AD) neuropathology is the progressive generation, aggregation and deposition of the 42 amino acid amyloid beta (Aβ42) peptide, and other related amyloidogenic molecules, into dense clumps of insoluble, pro-inflammatory senile plaque cores in the extracellular space of the brain. It is not generally appreciated that the Aβ42 peptide, derived via tandem beta-gamma secretase cleavage of the larger ~770 amino acid transmembrane beta-amyloid precursor protein (βAPP) is one of the “stickiest” peptides known, due in part to its high content of lipophillic and hydrophobic peptide domains (21.4% valine-isoleucine; Mager, 1998; Watson et al., 2005). Aβ42 peptide monomers have tremendously high potential for relatively rapid self-aggregation into Aβ42 peptide dimers and higher order fibrillar structures via long-range, non-covalent hydrophobic forces that over time promote β-pleated sheet conformations (Mager, 1998; Watson et al., 2005; Boutajangout and Wisniewski, 2013; Chang et al., 2013). There is abundant evidence that under normal, homeostatic conditions, Aβ42 peptide monomers and perhaps other higher order Aβ42 peptides are effectively “cleared” from the brain's extracellular medium by highly active and efficient innate-immune surveillance and phagocytic systems that limit excessive Aβ42 peptide dimerization, accumulation and further self-aggregation into pathological senile plaque lesions. Recently described, one of the key phagocytosis sensor-receptors responsible for Aβ42 peptide clearance from the human central nervous system (CNS) is very likely the triggering receptor expressed in myeloid/microglial cells 2 (TREM2) enriched in myeloid cells and microglial cells of the CNS (Benitez et al., 2013; Forabosco et al., 2013; Gonzalez Murcia et al., 2013; Guerreiro et al., 2013; Neumann and Daly, 2013; Zhao et al., 2013; Jones et al., 2014; Figure 1). This short paper is an update on some very recent observations on TREM2 neurobiology, on newly discovered roles for miRNA-34a-mediated signaling in human degenerative disease, including miRNA-34a-mediated effects on TREM2 expression, and how dysfunctional TREM2 signaling may contribute to amyloidogenesis in AD and in related progressive, inflammatory neurodegenerative diseases of the human CNS.

**Figure 1:**
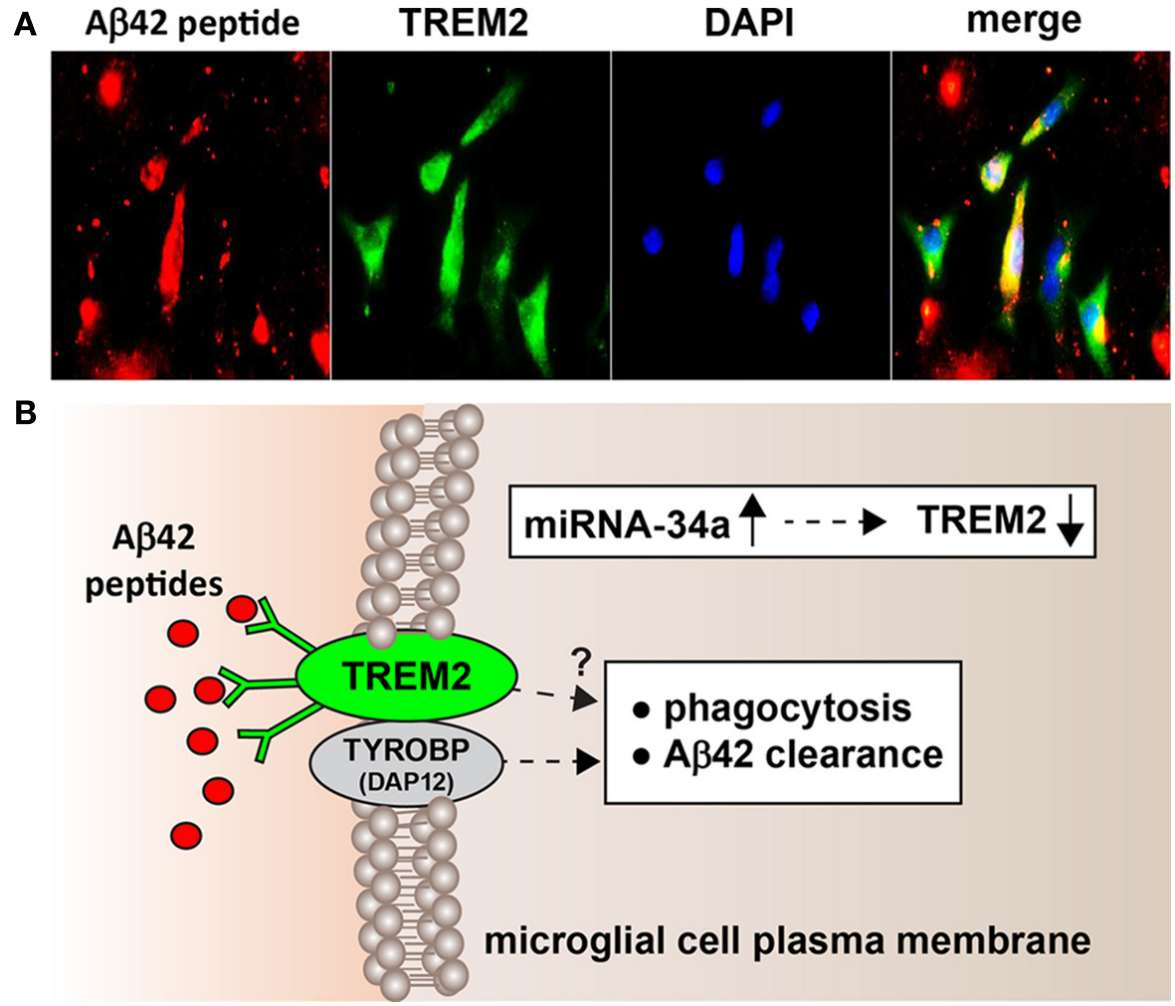
**(A)** Murine microglial cell mediated phagocytosis of Aβ42 peptides; 3 day old C8B4 murine microglia cells (ATCC CRL-2540; ATCC, Manassus VA) were treated with 5 μ M of Aβ42 for 24 h before staining; Aβ42 peptides (American Peptide Company, Sunnyvale, CA, cat # 62-0-80A) were prepared as described by Stine et al. (2003). Briefly, Aβ42 peptides were initially solubilized in hexafluoroisopropanol (HFIP; Fluka Chemical, cat# 52512; Sigma-Aldrich, St. Louis MO), aliquoted, and stored at −20°C as an HFIP film. After vacuum evaporation of HFIP, aliquoted peptide was re-suspended with DMSO to 5 mM and diluted to 5 μM into the cell culture media; cells were subsequently stained using a murine amyloid beta MABN10 (red fluorescence λ_max_ ~650 nm; anti-Aβ antibody, clone W0-2; Millipore, Bellerica MA), a TREM-2 antibody (M-227): sc-48765 (green fluorescence; λ_max_ ~510 nm; Santa Cruz, Santa Cruz CA) or DAPI nuclear stain; magnification 60×; note self-aggregation of Aβ42 peptide after 24 h and Aβ42 peptide affinity for TREM2 containing cells (leftmost panels) and internalization (rightmost panel; yellow merge; λ_max_ ~580 nm); additional relevant methods have been described (Griciuc et al., 2013; Zhao and Lukiw, 2013); **(B)** highly schematicized depiction of the possible actions of an NF-kB-regulated, miRNA-34a-mediated TREM2 sensor-phagocytosis protein down-regulated in AD brain; the triggering receptor for myeloid/microglial cells (TREM2) is a variably glycosylated transmembrane receptor known to be enriched in the microglial cell plasma membrane; signaling via the tyrosine kinase-binding protein (DNAX activation protein 12) [TYROBP (DAP12)] accessory receptor results in phagocytosis and ultimately, clearance of Aβ42 peptides (red ovals) from the extracellular space (Satoh et al., 2011; Benitez et al., 2013; Forabosco et al., 2013; Hickman and Khoury, 2014); interestingly, TREM2 knockout/knockdown mice have attenuated immunological and inflammatory responses and/or increases in age-related neuroinflammatory markers and cognitive deficiency (Jiang et al., 2013; Sieber et al., 2013); TYROBP knockout mice exhibit immune system deficits and an impairment in microglial cell differentiation (Nataf et al., 2005; Sieber et al., 2013); it is not clear what, if any, contribution TREM2 makes directly to phagocytosis and Aβ42 clearance (question mark) without TYROBP (DAP12); while no deficits in TYROBP (DAP12) have been observed in sporadic AD brain, insufficient TREM2 may be in part responsible for the inability to adequately phagocytose Aβ42 peptides, resulting in their buildup and self-aggregation in the extracellular space. Inset: miRNA-34a is found to be significantly increased in AD hippocampal CA1 and superior temporal lobe and in stressed microglial cells; miRNA-34a targeting of the TREM2 mRNA 3′-UTR appears to be in part responsible for this (see text); because miRNA-34a is encoded on an NF-kB-sensitive transcript, both anti-NF-kB and/or anti-miRNA strategies may be clinically useful in the restoration of homeostatic phagocytosis in the brain and CNS.

TREM2 (TREM-2, Trem2a), a variably glycosylated 230 amino acid type 1 transmembrane sensor-receptor protein of the immunoglobulin gene superfamily is translated from a ~1098 nucleotide (nt) linear mRNA transcribed at chr6p21.1, is highly expressed in microglial cells of the human CNS (NCBI GenBank NG_011561; BC032362; http://www.genecards.org/cgi-bin/carddisp.pl?gene=TREM2 Zhao et al., 2013; Jones et al., 2014). As a recently recognized myeloid/microglial cell surface phagocytosis sensor-receptor, TREM2 appears to play a critical function in immune surveillance, the sensing of extracellular debris and phagocytosis throughout the CNS, including the recognition and ingestion of neurotoxic Aβ42 peptides and other amyloidogenic extracellular debris (Benitez et al., 2013; Forabosco et al., 2013; Lattante et al., 2013; Neumann and Daly, 2013; Sieber et al., 2013; Hickman and Khoury, 2014; Jiang et al., 2014). TREM2 signaling is in part mediated through a tyrosine kinase-binding protein/DNAX activation adaptor protein of 12 kDa (TYROBP/DAP12; Sieber et al., 2013; Zhao et al., 2013; Hickman and Khoury, 2014; Figure 1). Deficiencies in TREM2 abundance and/or function are associated with a defective innate-immune system, bone fragility, deficits in phagocytosis and amyloidogenesis, neurological alterations leading to presenile dementia in the autosomal recessive, inflammatory neurodegenerative disorder polycystic lipomembraneous osteodysplasia with sclerosing leukoencephalopathy (PLOSL; MIM 221770; Jarvi-Hakola-Nasu disease), and more recently with Parkinson's disease (PD), AD and amyotrophic lateral sclerosis (ALS; Nataf et al., 2005; Satoh et al., 2011; Benitez et al., 2013; Forabosco et al., 2013; Guerreiro et al., 2013; Neumann and Daly, 2013; Sieber et al., 2013; Zhao et al., 2013; Abduljaleel et al., 2014; Cady et al., 2014). Conversely, acute brain injury-induced increases in TREM2 expression in microglia suggests that TREM2 may contribute to neurotrophic roles after brain ischemia and this may impart a long term neurological benefit in functional recovery (Kawabori et al., 2013; Abduljaleel et al., 2014). Genome-wide association studies and meta-analysis (GWAS/MA) for AD have recently identified an R47H (rs75932628) loss of function TREM2 variant as a significant risk factor for AD, conveying an increase for AD with an odds ratio of 1.3-8.8-fold (*p* = 0.008), an effect comparable to that of the *e4* allele of the 299 amino acid *APOE* lipid transporter (Gonzalez Murcia et al., 2013; Guerreiro et al., 2013; Neumann and Daly, 2013; Zhao et al., 2013). However, TREM2 R47H mutations appear to be relatively rare occurrences and may predispose only highly selective human populations to increased risk for age-related, pro-inflammatory neurodegenerative disorders such as AD (Gonzalez Murcia et al., 2013; Guerreiro et al., 2013; Hampel and Lista, 2013; Lattante et al., 2013; Bagyinszky et al., 2014). Indeed, AD cases are each highly complex, heterogeneous and multigenic neurodegenerative disorders and can be divided into those with a genetic or familial component (~5% of all cases) or a sporadic or idiopathic form of AD with no known genetic cause (95% of all cases; Blennow et al., 2006; Lukiw, 2013b; Bagyinszky et al., 2014). AD can be further classified as either early onset (under 65 years of age), or late-onset AD (LOAD; over 65 years of age; Bagyinszky et al., 2014; Rosenthal and Kamboh, 2014). Evidence for the loss of function R47H mutation remains extremely rare in late onset AD in diverse human populations including relatively large, recent studies in Chinese Han (*N* = 625) or Japanese (*N* = 4688) groups (Ma et al., 2014; Miyashita et al., 2014; Rosenthal and Kamboh, 2014). Indeed, much recent, independently derived data support the contention that the incidence of genetic mutations and epigenetic factors in AD varies widely amongst different human populations with different evolutionary backgrounds (Raj et al., 2010; Olson, 2012; Lukiw, 2013a,b). Interestingly, genetic-based loss-of-function mutations in TREM2 in LOAD may have the same end effect as a deficiency of a functional TREM2 in sporadic AD, with both pathways leading to compromised TREM2-mediated signaling and defective Aβ42 sensing and removal by phagocytosis.

MicroRNAs (miRNAs) are small non-coding, single stranded RNAs (ssRNAs) that currently represent the smallest known carriers of highly selective genetic regulatory information in the human CNS (Lukiw, 2007; Hill et al., 2014a,b; Maffioletti et al., 2014). As highly mobile, autonomous genetic elements abundant in brain cell cytoplasm, the cerebrospinal fluid (CSF) and in the systemic circulation, miRNAs may be diagnostic for AD and other human CNS diseases (Alexandrov et al., 2012; Dorval et al., 2013). The primary mode of miRNA action is to recognize and bind to complementary ribonucleotide sequences in the 3-prime un-translated region (3′-UTR) of target messenger RNAs (mRNAs) and in doing so, down-regulate their expression (Lagos-Quintana et al., 2001; Lukiw et al., 2008; Guo et al., 2010). Several independent laboratories have recently reported the highly selective up-regulation of specific pathology-related miRNAs in AD brain including: **(1)** a significant up-regulation in the pro-inflammatory miRNA-34a (encoded at chr1p36.22) in AD and in Aβ42 peptide- and cytokine-stressed human primary brain cells (Cogswell et al., 2008; Lukiw, 2012; Zhao et al., 2013); **(2)** a significant miRNA-34a up-regulation in amyloid overexpressing transgenic mouse models of AD (Wang et al., 2009; Zhao et al., 2013); **(3)** a productive and CNS-relevant miRNA-34a-TREM2-3′-UTR interaction resulting in TREM2 deficits in cellular models of AD (Zhao and Lukiw, 2013; Zhao et al., 2013; Jones et al., 2014); and **(4)** an NF-kB-mediated up-regulation of miRNA-34a coupled to a down-regulation of TREM2 in human neuronal-glial co-cultures (Alexandrov et al., 2013). The miRNA-34a-mediated down-regulation of TREM2 appears to be due to an unusually strong miRNA-34a recognition feature within the 299 nt TREM2 mRNA 3′-UTR region (energy of association, E_A_ <-16 kcal/mol; http://www.genecards.org/cgi-bin/carddisp.pl?gene=TREM2; Zhao et al., 2013; Abduljaleel et al., 2014; Jiang et al., 2014; Jones et al., 2014). Interestingly, the stress- and inflammation-induced transcription factor NF-kB, a driver for miRNA-34a expression, is also selectively up-regulated in AD-affected brain regions, and both NF-kB inhibitors and stabilized anti-miRNA-34a appear to be effective in restoring TREM2 back to homeostatic levels, at least in *in vitro* primary cell models of inflammatory neurodegeneration (Crampton and O'Keeffe, 2013; Lukiw, 2013a; Zhao and Lukiw, 2013; Zhao et al., 2013). Pathologically up-regulated miRNA-34a-signaling has also been recently associated **(1)** with the repression of expression of several selected genes involved in cell survival and oxidative defense pathways such as Bcl2 and SIRT1 (Bhatnagar et al., 2014); **(2)** with spinal cord tissues in ALS (Cady et al., 2014); **(3)** with altered immunity associated with multiple sclerosis (Junker et al., 2009); **(4)** with progressive neurotrophic deficits including dysfunctional Bcl-2 signaling in transgenic murine models of AD (Wang et al., 2009); **(5)** with altered synaptogenesis (Agostini et al., 2011); **(6)** with deficient immune and phagocytotic responses in progressive inflammatory degeneration in cardiovascular disease (Boon et al., 2013); **(7)** with aging of the murine brain (Li et al., 2011); **(8)** with vasculature aging and cellular senescence (Boon et al., 2013; Agostini and Knight, 2014); **(9)** with blood mononuclear cells in sporadic AD patients (Schipper et al., 2007; Bhatnagar et al., 2014); **(10)** with lower mini-mental state examination (MMSE) scores when detected in the blood plasma of AD patients (Bhatnagar et al., 2014) and **(11)** with the progressive inflammatory neurodegeneration and epileptiform activities associated with epilepsy and the early stages of AD (Zhao et al., 2013; Henshall, 2014). The role of the CNS-enriched miRNA-34a and other pro-inflammatory miRNAs in epilepsy and AD is particularly interesting due to the overlapping neuropathology of these two neurological disorders with respect to the incidence of seizures and cognitive decline first apparent in the earliest stages of each disease (Vossel et al., 2013; Hill et al., 2014a,b).

Strengthening evidence continues to support the hypothesis that multiple genes, through multiple genetic processes, drive the initiation, propagation and course of sporadic AD. Epigenetic mechanisms involving NF-κB-mediated, miRNA-34a up-regulation and consequent down-regulation of TREM2 expression may drive the progressive extinction of the phagocytic response that in turn contributes to dysfunctional innate-immunity, amyloidogenesis and inflammatory neurodegeneration. Current data also suggest that the orchestrated interaction of at least two independent gene products on two different chromosomes—miRNA-34a at chr1p36.22 and TREM2 at chr6p21.1—modulate TREM2 activities, the sensing of potentially hazardous waste molecules in the extracellular space, and the phagocytosis and clearance of this neurotoxic debris to maintain functional homeostasis in the CNS. Importantly, defective regulation of miRNA-34a and TREM2 signaling and other epigenetic effects on gene expression in sporadic AD would not be detectable via classical GWAS/MA or single nucleotide polymorphism (SNP) analysis of the genome (Hampel and Lista, 2013; Lukiw, 2013b). While dysfunction along the miRNA-34a-TREM2-TYOBP(DAP12) axis may be a particularly strong contributor to phagocytosis deficits and amyloidogenesis in AD it is important to note that other miRNA-mRNA pairings may also be involved in Aβ42 clearance and altered innate-immune responses in this complex genetic regulatory network. AD-relevant stress-mediated increases in miRNA-34a in cultured brain cells, subsequent down-regulation in the expression of TREM2-3'-UTR reporter vectors, and rescue by anti-NF-kB or anti-miRNA-34a pharmacological strategies indicates that TREM2 and accessory genetic signaling components that drive defective Aβ42 peptide sensing and phagocytosis can be effectively quenched, at least in *in vitro* studies (Lukiw, 2013b; Zhao et al., 2013; Jones et al., 2014). There is currently a great deal of pharmacological interest in the use of miRNA-34a mimics and their potential role in treating degenerative disease, including CNS disease and cancer, and miRNA-34a mimics have become the first half-life stabilized miRNAs to reach phase 1 clinical trials (Boon et al., 2013; Zhao et al., 2013; Agostini and Knight, 2014). Indeed anti-NF-kB, anti-miRNA-34a and/or analogous pharmacological molecular strategies may be useful in the future clinical management of AD and other multi-pathway neurological diseases with an amyloidogenic component, including novel combinatorial therapeutic approaches that have not yet been considered.

## Conflict of interest statement

The authors declare that the research was conducted in the absence of any commercial or financial relationships that could be construed as a potential conflict of interest.
